# Comparison of serum protein profiles between major depressive disorder and bipolar disorder

**DOI:** 10.1186/s12888-020-02540-0

**Published:** 2020-04-03

**Authors:** Sang Jin Rhee, Dohyun Han, Yunna Lee, Hyeyoung Kim, Junhee Lee, Kangeun Lee, Hyunsuk Shin, Hyeyoon Kim, Tae Young Lee, Minah Kim, Se Hyun Kim, Yong Min Ahn, Jun Soo Kwon, Kyooseob Ha

**Affiliations:** 1 Department of Neuropsychiatry, Seoul National University Hospital, Seoul, Republic of Korea; 2 Department of Psychiatry, Seoul National University College of Medicine, Seoul, Republic of Korea; 3 Proteomics Core Facility, Biomedical Research Institute, Seoul National University Hospital, Seoul, Republic of Korea; 4 Department of Psychiatry, Inha University Hospital, Incheon, Republic of Korea; 5 Department of Pathology, Seoul National University College of Medicine, Seoul, Republic of Korea; 6 Institute of Human Behavioral Medicine, Seoul National University Medical Research Center, Seoul, Republic of Korea; 7 Department of Neuropsychiatry, Pusan National University Yangsan Hospital, Yangsan, Republic of Korea

**Keywords:** Major depressive disorder, Bipolar disorder, Proteomics, rab7 protein, ROCK2 protein, human, XPO7 protein, human

## Abstract

**Background:**

Major depressive disorder and bipolar disorder are prevalent and debilitating psychiatric disorders that are difficult to distinguish, as their diagnosis is based on behavioural observations and subjective symptoms. Quantitative protein profile analysis might help to objectively distinguish between these disorders and increase our understanding of their pathophysiology. Thus, this study was conducted to compare the peripheral protein profiles between the two disorders.

**Methods:**

Serum samples were collected from 18 subjects with major depressive disorder and 15 subjects with bipolar disorder. After depleting abundant proteins, liquid chromatography-tandem mass spectrometry (LC-MS/MS) and label-free quantification were performed. Data-dependent acquisition data were statistically analysed from the samples of 15 subjects with major depressive disorder and 10 subjects with bipolar disorder who were psychotropic drug-free. Two-sided *t*-tests were performed for pairwise comparisons of proteomes to detect differentially-expressed proteins (DEPs). Ingenuity Pathway Analysis of canonical pathways, disease and functions, and protein networks based on these DEPs was further conducted.

**Results:**

Fourteen DEPs were significant between subjects with major depressive disorder and those with bipolar disorder. Ras-related protein Rab-7a (t = 5.975, *p* = 4.3 × 10^− 6^) and Rho-associated protein kinase 2 (t = 4.782, *p* = 8.0 × 10^− 5^) were significantly overexpressed in subjects with major depressive disorder and Exportin-7 (t = -4.520, *p* = 1.5 × 10^− 4^) was significantly overexpressed in subjects with bipolar disorder after considering multiple comparisons. Bioinformatics analysis showed that cellular functions and inflammation/immune pathways were significantly different.

**Conclusions:**

Ras-related protein Rab-7a, Rho-associated protein kinase 2, and Exportin-7 were identified as potential peripheral protein candidates to distinguish major depressive disorder and bipolar disorder. Further large sample studies with longitudinal designs and validation processes are warranted.

## Background

Major depressive disorder (MDD) and bipolar disorder (BD) are both prevalent psychiatric disorders with an overall prevalence of 3–10% and 2–4%, respectively [1, 2]. Both disorders are debilitating, as MDD (unipolar depressive disorder) and bipolar affective disorder were ranked 1st and 4th among all mental and substance use disorders when calculating the Disability-Adjusted Life Year value in the Korean population [3]. Moreover, their associated mortality rates including death from suicide have significantly increased [4].

Distinguishing MDD from BD is challenging because diagnosis is mostly based on behavioural observations and subjective symptoms, and the conditions show similar manifestations. Although there is accumulating research regarding the differences between MDD and BD [5], more than 30% of subjects with BD are misdiagnosed initially with MDD [6], and the average lag time for correctly diagnosing BD after a diagnosis of MDD is nearly 10 years [7]. This might lead to mistreatment and antidepressant monotherapy prescription for subjects with BD [8], which worsens patient outcome by inducing hypomanic/manic or mixed states during the course of the disorder [9].

As the diagnosis of psychiatric disorders can be somewhat subjective, there has been growing efforts to discover objective biomarkers. Previous studies have involved large-scale genetic analyses followed by transcriptome evaluations [10, 11]. Although these studies have shown some promising results, they also demonstrated the importance of focusing on -omics that are more reflective of the functions and phenotypes of an individual [12, 13]. Traditionally, studies have focused on the central nervous system (post-mortem brain tissues and cerebrospinal fluid), but this is practically challenging in clinical practice because of invasiveness and accessibility issues [13, 14].

Thus, studies to quantitatively measure peripheral protein profiles that are associated with mood disorders have increased [12–14]. Analysing the protein profiles of BD and differentiating them from those of MDD, in particular, can increase the understanding of disease pathophysiology and help us to distinguish these disorders. Because of advances in analysis techniques, it is now possible to simultaneously measure numerous proteins in individual samples [12]. Different articles have reported quantitative protein profile differences between MDD or BD and controls [12, 15–18]. However, few studies have compared the profiles of MDD and BD [19, 20]. Thus, this study was conducted to compare the peripheral proteomic profiles between MDD and BD.

## Methods

### Clinical samples

Serum samples were initially collected from 18 subjects with MDD and 15 with BD (4 BD-I, 10 BD-II, and 1 BD NOS), aged 16–42 years, from Seoul National University Hospital and Seoul National University Bundang Hospital between May 2012 and September 2017. Subjects were diagnosed clinically based on the DSM-IV or DSM-5 diagnosis for MDD and BD by psychiatric specialists. No subjects were diagnosed with substance-related (alcohol or drug use) disorders, and no subjects suffered from physical illnesses such as hypertension, endocrine diseases including diabetes or thyroid diseases, hypercholesteremia, liver diseases, or epilepsy. Those showing evidence of mental retardation or organic brain disease or with difficulty interpreting the Korean language were excluded. Demographic data were collected with written questionnaires, and symptom severity was assessed with the Hamilton depression rating scale [21]. Final statistical analysis was conducted for 25 subjects who had not been administered psychotropic drugs for at least 2 weeks and had no missing values for demographic and clinical data. At the time of blood collection, all subjects with MDD were in a mild-to-moderate depressive state and the clinical states of the subjects with BD were depressed (*N* = 5), depressive with irritability (*N* = 4), or hypomanic (*N* = 1).

Written informed consent was obtained from all individuals. Informed consent from both the individuals and parents/guardians was obtained for those under the age of 18. The study protocol was approved by the Institutional Review Board of Seoul National University Hospital (IRB No. 1704–075-846) and was conducted in accordance with the Declaration of Helsinki.

### Serum sample preparation

Blood was collected in the morning after overnight fasting for at least 8 h. Blood samples were centrifuged at 3000 rpm at 4 °C for 10 min, and the serum was collected and stored at < − 70 °C. Serum samples were prepared according to the method reported by Geyer et al. (2016) [22] with some modifications. The protein digestion process was optimised to 2 μL of each serum sample. Briefly, digestion buffer (8 M urea, 5 mM TCEP, 20 mM CAA in 0.1 M ABC) was added to the serum sample. The mixture was boiled for 25 min at 60 °C to denature and alkylate the proteins. After cooling to room temperature, protein digestion was performed at 37 °C overnight using a trypsin/LysC mixture at a 100:1 protein-to-protease ratio. The second digestion was performed at 37 °C for 2 h using trypsin (enzyme-to-substrate ratio [w/w] of 1:1000). All resulting peptides were acidified with 10% trifluoroacetic acid and desalted using homemade C18-StageTips as described [23, 24]. Desalted samples were completely dried with a vacuum dryer and stored at − 80 °C.

### Establishment of a matching spectral library

To construct a matching spectral library for matching between runs [25], a MARS-14 column (Agilent Technologies, Santa Clara, CA, USA) was used to remove the 14 blood proteins of highest abundance according to the manufacturer’s instructions. The depleted samples were digested using the 2-step filter-aided sample preparation as described previously [23, 24]. Digested peptides were desalted using homemade C18-StageTips. For the in-depth data set, 25 μg of purified peptides were fractionated using an Agilent 1260 bioinert HPLC (Agilent Technologies) equipped with an analytical column (4.6 × 250 mm, 5-μm particle). High-pH reversed-phase peptide fractionation was performed at a flow rate of 0.8 mL/min over a 60-min gradient using solvent A (15 mm ammonium hydroxide in water) and solvent B (15 mM ammonium hydroxide in 90% acetonitrile). A total of 96 fractions was collected each minute and non-contiguously pooled into 24 fractions. The fractions were dried in a vacuum centrifuge and stored at − 80 °C until liquid chromatography-tandem mass spectrometry (LC-MS/MS) analysis.

### LC-MS/MS analysis

LC-MS/MS analysis methods were performed using Quadrupole Orbitrap mass spectrometers, Q-exactive plus (Thermo Fisher Scientific, Waltham, MA, USA) coupled to an Ultimate 3000 RSLC systems (Dionex, Sunnyvale, CA, USA) with a nano electrospray source as previously described with some modifications [23, 26]. Peptide samples were separated on the 2-column setup with a trap column (75 μm I.D. × 2 cm, C18 3 μm, 100 Å) and an analytical column (50 μm I.D. × 15 cm, C18 1.9 μm, 100 Å). Prior to sample injection, the dried peptide samples were redissolved in solvent A (2% acetonitrile and 0.1% formic acid). After the samples were loaded onto the nano LC, a 90-min gradient from 8 to 30% solvent B (100% acetonitrile and 0.1% formic acid) was applied to all samples. The spray voltage was 2.0 kV in positive ion mode and the temperature of the heated capillary was set to 320 °C. Mass spectra were acquired in data-dependent mode using a top 15 method on a Q Exactive. The Orbitrap analyser scanned precursor ions with a mass range of 300–1650 m/z and resolution of 70,000 at m/z 200. Higher-energy collisional dissociation (HCD) scans were acquired on the Q Exactive at a resolution of 17,500. HCD peptide fragments were acquired at a normalised collision energy of 28. The maximum ion injection times for the survey and MS/MS scans were 20 and 120 ms, respectively.

### Data processing for label-free quantification

MaxQuant (version 1.5.3.1) was used to process mass spectra [27], and the Andromeda engine was used to match MS/MS spectra with the Human Uniprot protein sequence database (December 2014, 88,657 entries) [28]. For total protein level analysis, primary searches were conducted using a 6-ppm precursor ion tolerance. The settings were as follows; MS/MS ion tolerance at 20 ppm, *N*-Acetylation of proteins and oxidation of methionine as variable modifications, cysteine carbamido-methylation as fixed modification, and enzyme specificity to full tryptic digestion [26]. Peptides with a minimal length of six amino acids, and up to two missed cleavages were considered, and the false discovery rate (FDR) was set to 1% at peptide, protein, and modification levels [26]. To maximize quantification events across samples, matching between runs was performed using the depleted sample as a library.

### Statistical analysis

Among the 33 subjects, 25 were free of psychotropic drugs for at least 2 weeks and had no missing values for demographic and clinical data. Data-dependent acquisition (DDA) data were statistically analysed in these subjects to enable comparisons of demographic and clinical characteristics between the two diagnostic groups and analyse the proteomic profiles independently of the effects of psychotropic drugs. Demographic and clinical differences between the diagnostic groups were analysed using a Mann-Whitney U-test for continuous variables and Fisher’s exact test for dichotomous variables.

Statistical analyses for the DDA data were performed using Perseus software [29]. Initially, proteins identified as only identified by site, reverse, and contaminants were removed. The expression level of proteins in the serum was estimated by determining their Intensity Based Absolute Quantification (iBAQ) values calculated using Maxquant software. Because of the skewed distribution of the data, log_2_ transformation was conducted for these values. Valid values were filtered with proteins with a minimum of 70% quantified values in at least one diagnostic group. Missing values of the proteins were imputed based on a normal distribution (width = 0.3, down-shift = 1.8) to simulate signals of low-abundance proteins. Two-sided *t*-tests were performed for pairwise comparisons of proteomes to detect differentially-expressed proteins (DEPs). The protein abundances were subjected to z-normalisation followed by hierarchical clustering with Pearson’s correlation distance.

Canonical pathways, diseases and functions, and protein networks were evaluated by Ingenuity Pathway Analysis (IPA, QIAGEN, Hilden, Germany) [30] based on the annotated DEPs with matched gene names. The analytical algorithms embedded in Ingenuity Pathway Analysis uses lists of DEPs to predict biological processes and pathways. Additionally, a tree-map was visualised, in which the major boxes represented categories of related biological functions or diseases. Statistical significance of both the gene ontology classification and enrichment analysis was determined by the Fisher’s exact test. All statistical tests were two-sided and *p* < 0.05 was considered statistically significant.

## Results

### Demographic and clinical characteristics

In total, 15 subjects with MDD and 10 subjects with BD were included in the final statistical analysis of the DDA data. There were no significant differences between diagnostic groups regarding age, sex, body mass index, smoking, duration from onset, and duration from the first psychotropic treatment. Depressive symptoms were more severe in the MDD group (HAMD score; 15.33 ± 4.61 vs 12.10 ± 3.54, *p* = 0.02). A summary of the characteristics is presented in Table 1.

**Table 1:** Demographic and clinical characteristics of the study subjects

Characteristics	Major depressive disorder (*n* = 15)	Bipolar disorder (*n* = 10)	Statistics	*p*-value
Age, mean ± SD, years	28.53 ± 8.04	25.10 ± 4.91	Z = −1.059	0.29
BMI, mean ± SD, kg/m^2^	22.42 ± 5.18	22.41 ± 5.17	Z = −0.055	0.96
HAMD, mean ± SD	15.33 ± 4.61	12.10 ± 3.54	Z = −2.320	0.02
Sex (Female), *n* (%)	10 (66.7%)	7 (70.0%)	Fisher’s exact test	> 0.99
Current smoker, *n* (%)	1 (6.7%)	3 (30.0%)	Fisher’s exact test	0.27
Duration from onset, mean ± SD, years	3.73 ± 4.57	4.80 ± 2.94	Z = −1.654	0.10
Duration from the first psychotropic treatment, mean ± SD, years	0.33 ± 0.49	1.90 ± 3.28	Z = −0.974	0.33

Abbreviations: *SD* standard distribution, *BMI* body mass index, *HAMD* Hamilton depression rating scale

### Quantitative analysis

First, we performed comprehensive serum profiling using pooled serum samples with the depletion of 14 highly-abundant proteins to generate a peptide matching library, which consisted of 1616 proteins and 16,505 peptides. Thirty-three serum samples were analysed by unbiased single-shot approaches and “match between run” functionality with the constructed peptide library. We quantified 481 serum proteins with at least two peptides. After considering proteins that were quantified by at least 70% in either the MDD group or BD group from drug-free patients, 268 proteins were subjected to statistical analysis (Additional file 1).

**Fig. 1:**
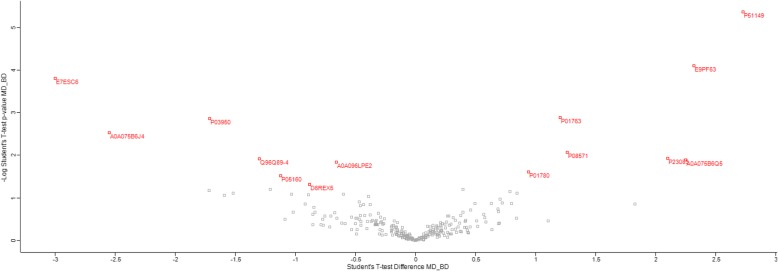
Volcano plot of *p*-values for differentially-expressed proteins between major depressive disorder and bipolar disorder. Representative protein IDs in red indicate statistically significant differentially-expressed proteins based on a *p* value < 0.05

**Fig. 2:**
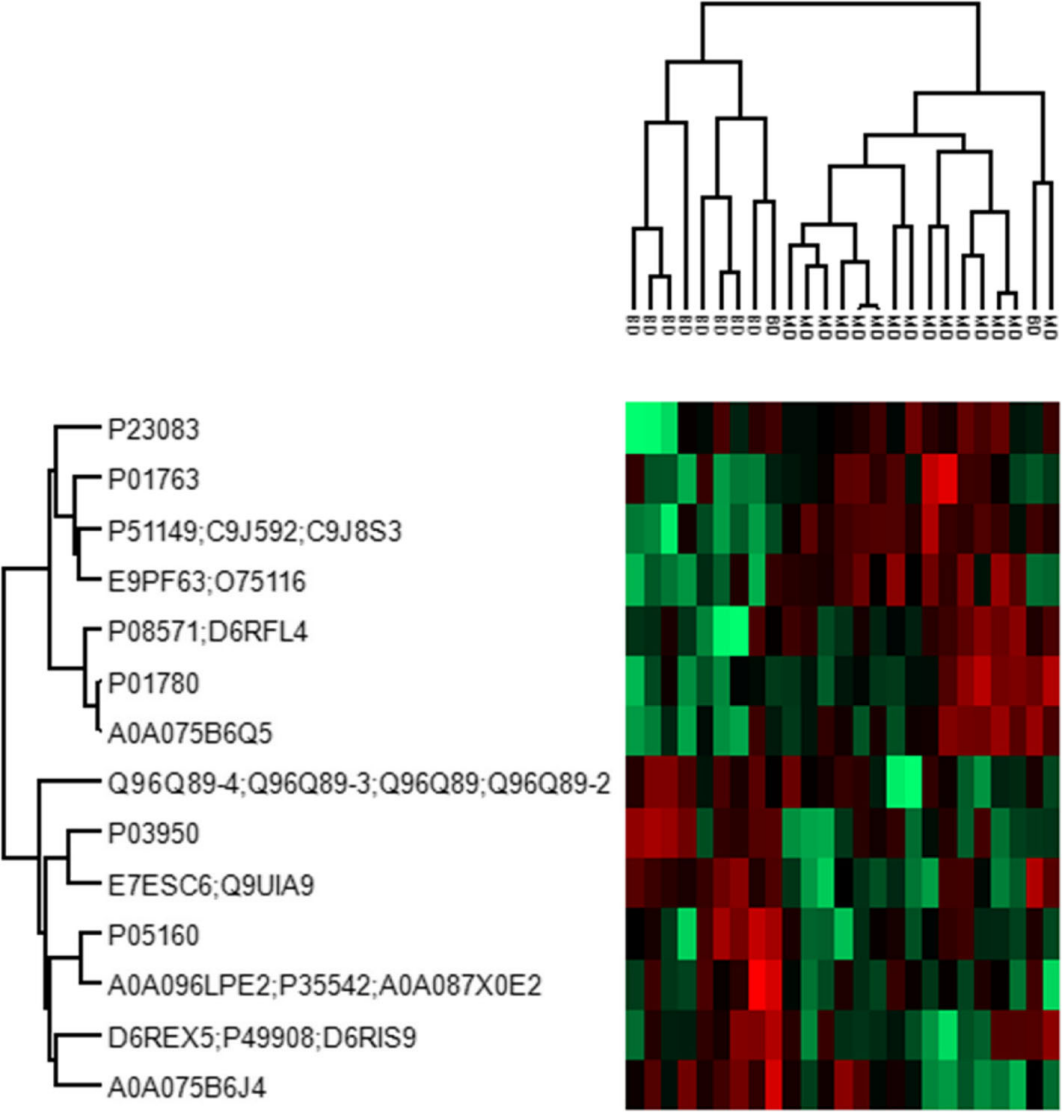
Hierarchical clustering of statistically significant differentially-expressed proteins

Two-sample *t*-tests showed that the levels of 14 proteins were significantly different between MDD and BD (Fig. 1). When adjusting for multiple comparisons using the Benjamini-Hochberg FDR adjusted *p*-value, the levels of Ras-related protein Rab-7a (RAB7A), Rho-associated protein kinase 2 (ROCK2), and Exportin-7 (XPO7) were still significantly different (Table 2 and Additional file 2). Hierarchical clustering revealed that the two disorders generally clustered together (Fig. 2).

**Table 2:** Differentially expressed proteins between major depressive disorder and bipolar disorder

Majority protein IDs	Protein names	Gene names	Fold change^a^	Student’s t-test statistics (t)	p-value^b^
Overexpressed in MDD					
P51149;C9J592;C9J8S3	Ras-related protein Rab-7a	*RAB7A*	2.729	5.975	4.3 * 10^**−6**^
E9PF63;O75116	Rho-associated protein kinase 2	*ROCK2*	2.321	4.782	8.0 * 10^**−5**^
P01763	Ig heavy chain V-III region WEA	*IGHV3–48*	1.205	3.668	0.001
P08571;D6RFL4	Monocyte differentiation antigen CD14;Monocyte differentiation antigen CD14, urinary form;Monocyte differentiation antigen CD14, membrane-bound form	*CD14*	1.266	2.875	0.009
P23083	Ig heavy chain V-I region V35	*IGHV10R15–1*	2.103	2.739	0.011
A0A075B6Q5	Ig heavy variable 3–64	*IGHV3–64*	2.252	2.698	0.013
P01780	Ig heavy chain V-III region JON	*IGHV3–7*	0.941	2.404	0.025
Overexpressed in BD					
E7ESC6;Q9UIA9	Exportin-7	*XPO7*	−2.999	−4.520	1.5 * 10^**−4**^
P03950	Angiogenin	*ANG*	−1.714	−3.636	0.001
A0A075B6J4	Ig lambda variable 3–25	*IGLV3–25*	−2.550	−3.326	0.003
Q96Q89–4;Q96Q89–3;Q96Q89;Q96Q89–2	Kinesin-like protein KIF20B	*KIF20B*	−1.298	−2.718	0.012
A0A096LPE2;P35542;A0A087X0E2	Serum amyloid A-4 protein; Serum amyloid A protein	SAA2-SAA4;*SAA4*	−0.660	−2.640	0.015
P05160	Coagulation factor XIII B chain	*F13B*	−1.120	−2.315	0.030
D6REX5;P49908;D6RIS9	Selenoprotein P	*SEPP1;SELENOP*	−0.881	−2.088	0.048

Abbreviations: *MDD* major depressive disorder, *BD* bipolar disorder

^a^Fold change calculated by the difference of the logarithmic_(2)_ transferred intensity

^b^Boldface *p*-values are significant when adjusting for multiple comparison by using the Benjamini-Hochberg FDR adjusted *p*-value

### Bioinformatics analysis

**Fig. 3:**
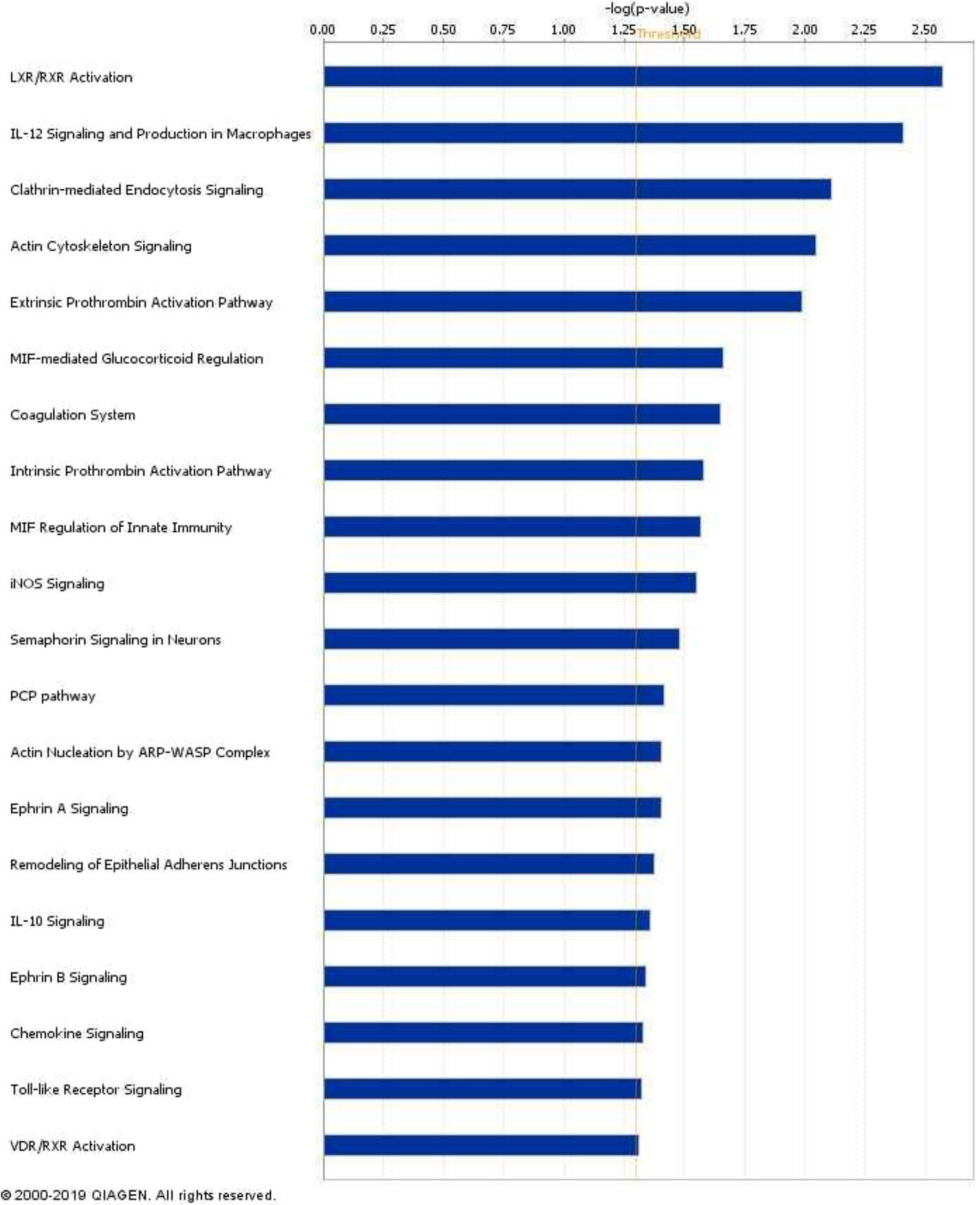
Canonical pathway analysis of the differentially-expressed proteins

The 14 proteins that were differentially expressed were subjected to bioinformatics analysis. The top five significant canonical pathways were LXR/RXR Activation (CD14, SAA4), IL-12 Signalling and Production in Macrophages (RAB7A, SAA4), Clathrin-mediated Endocytosis Signalling (RAB7A, SAA4), Actin Cytoskeleton Signalling (ROCK2, CD14), and the Extrinsic Prothrombin Activation Pathway (F13B). RAB7A was also included in the Remodeling of epithelial adherens junction pathway, and ROCK2 was included in the following pathways, Semaphorin Signalling in Neuron, PCP pathway, Actin Nucleation by ARP-WASP Complex, Ephrin A Signalling, Ephrin B Signalling, and Chemokine Signalling (Fig. 3 and Additional File 3).

**Fig. 4:**
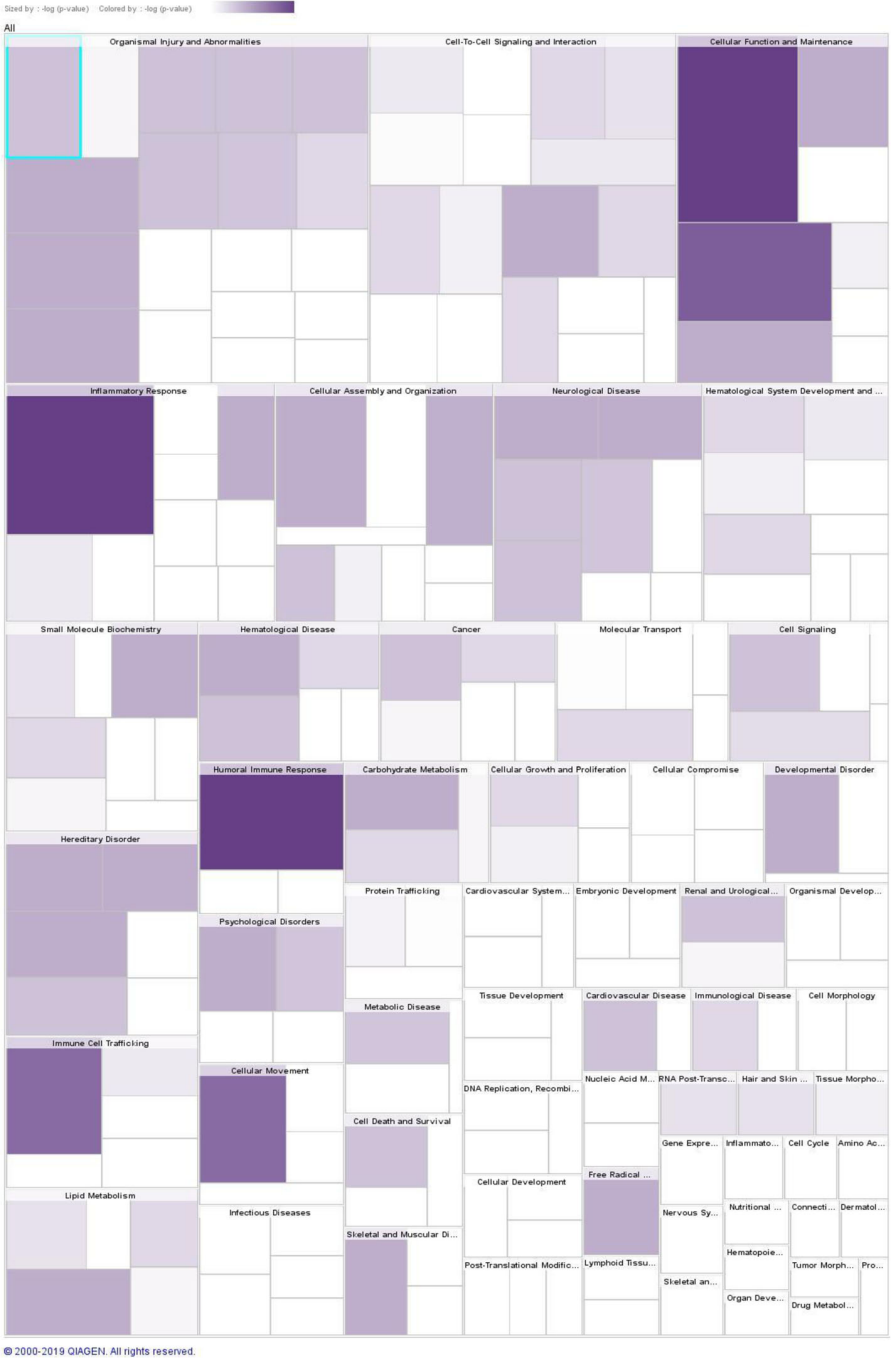
Tree-map for diseases and functions of the differentially-expressed proteins. Major boxes represent categories of biological functions/diseases and individual rectangles represent an individual biological function or disease. The size of a rectangle is correlated with increasing overlap significance and darker colours indicate lower p-values

**Fig. 5:**
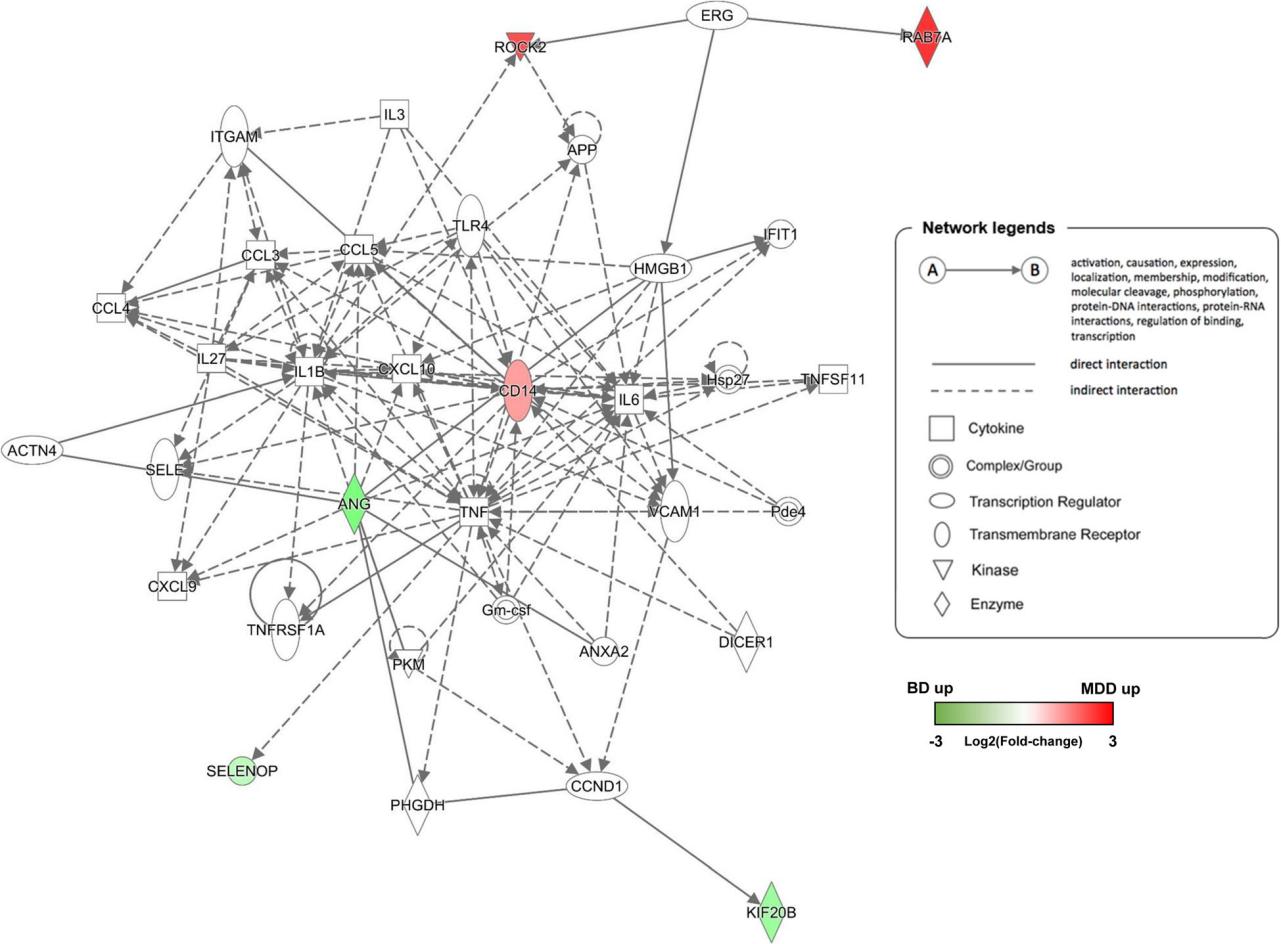
Top protein network generated by IPA for 14 differentially-expressed serum proteins in MDD versus BD. Direct and indirect interactions are represented by the solid and dashed lines, respectively. The shapes represent the molecular classes of the proteins defined in the legend. The protein interaction networks were generated through the use of IPA. MDD, major depressive disorder; BP, bipolar disorder; IPA, Ingenuity Pathway Analysis

The tree-map for diseases and functions is shown in Fig. 4. The DEPs were particularly enriched in organismal injury and abnormalities, cell-to cell signalling and interaction, cellular function and maintenance, inflammatory response, cellular assembly and organization, and neurological disease. (Additional File 3). Finally, network analysis of 14 DEPs in the serum of MDD samples versus BD were assessed. The first network consisted of six proteins (RAB7A, ROCK2, CD14, ANG, SELENOP, and KIF20B), and its related diseases and functions incorporated cellular movement, haematological system development and function, and immune cell trafficking (Fig. 5).

## Discussion

In this study, we detected 14 DEPs between psychotropic drug-free MDD and BD subjects. RAB7A and ROCK2 were significantly overexpressed in MDD and XPO7 was significantly overexpressed in BD even after multiple comparisons. Bioinformatics analysis showed that cellular functions and inflammation/immune response pathways were significantly different.

To date, there are two published articles directly comparing DEPs between MDD and BD [19, 20]. Chen et al. (2015) compared plasma samples from drug-naïve patients and identified 25 DEPs, whereas Ren et al. (2017) compared plasma samples from drug-free patients and identified nine DEPs [19, 20]. However, no proteins were common between studies [19, 20]. We compared serum samples from subjects with MDD and BD and found only one protein, Rho-associated protein kinase 2 (ROCK2), as a duplicate protein from the previous studies [20]. These discrepancies are likely related to variations in the study designs and differences in the demographic and clinical characteristics of the study. Our study was based on serum samples, and there is evidence that expression between the serum and plasma profiles can differ for certain proteins [18], which might explain these discrepancies. Moreover, the overall depressive symptoms were milder in our study, which might have also led to differences in the peripheral profiles.

The most significant DEP based on the fold-change and *p*-value was RAB7A, which was overexpressed in MDD compared to levels in BD. This protein is a key regulator of endo-lysosomal trafficking [31] and is distributed in various components of the body including the central nervous system and peripheral blood [32, 33]. The level of RAB7A was increased in the cerebrospinal fluid of subjects with Alzheimer disease [33], and a recent study demonstrated that RAB7A gene expression was significantly changed in both the hippocampus and peripheral blood of subjects with Alzheimer disease, suggesting the possibility of screening Alzheimer disease from the peripheral blood [32]. However, its association with mood disorders is not well-understood. A previous study showed that RAB7A gene expression in the post-mortem brain was up-regulated in patients with depression who died by suicide [34]. Recently, RAB7A was demonstrated to modulate endoplasmic reticulum stress [35], which is associated with mood disorders [36, 37]. Other functions of RAB7A include growth-factor-mediated cell signalling and lipid metabolism [38], which are also proposed mechanisms involved in the pathophysiology of mood disorders [39, 40]. Further studies focusing on the association between RAB7A and the different manifestations of mood disorders based on these functions are needed.

ROCK2 was also overexpressed in MDD compared to in BD. The direction of expression and the protein itself were consistent with the results found by Chen et al. [20]. ROCK is a serine/threonine kinase that is a crucial regulator of the actin cytoskeleton and cell polarity [41]. It has two highly homologous isoforms, ROCK1 and ROCK2; interestingly ROCK2 is distributed mostly in the brain, spinal cord, and heart [42]. Inhibition of ROCK increases neurite outgrowth and axonal regeneration and activates pro-survival protein kinase B in the central nervous system, and its potential for treating neurodegenerative diseases is being explored [41]. Direct associations considering the expression of ROCK2 and mood disorders are limited; one report showed that placental ROCK2 is down-regulated in women with depression [43]. However, there is evidence that ROCK2 expression increases during sleep deprivation [44] and ROCK2 is involved in the circadian variation of vascular contractility [45]. There is increasing evidence of the differences in sleep abnormalities and circadian rhythm dysregulation between MDD and BD, and circadian rhythm dysregulation has also been proposed as a marker specific for BD compared to MDD [46]. ROCK2 might also help to distinguish MDD from BD; studies of its role in circadian rhythm and mood dysregulation are needed.

XPO7 mediates the nuclear export of proteins with broad substrate specificity and was identified as one of the significant genes in a genome-wide association study on alcohol dependence [47, 48]. In a confirmation study, this gene was significantly changed in patients with BD with comorbid alcohol dependence, but not in those without alcohol dependence [49]. However, its mechanism in alcohol dependence or mood disorders is unclear because of the lack of current literature linking XPO7 function with psychiatric manifestations.

Bioinformatics analysis revealed that cellular functions and inflammation/immune pathways are significantly altered. The most significant canonical pathway was found to be LXR/RXR (liver X receptor/retinoic acid receptor) activation. LXRs form heterodimers with RXRs, which regulate the expression of genes controlling sterol/fatty acid metabolism/homeostasis [50]. Interestingly, this pathway was recently demonstrated to be significantly altered in plasma DEPs in a mouse model of depression [51]. Inflammation and immune pathways were also significantly different, and these have been proposed to be involved in mood disorders [52–54]. Analysing the differences in protein expression associated with certain biological pathways, and mood disorder subtypes might improve our ability to differentiate BD from MDD.

The present study had several limitations. First, the sample size was small, and the study lacked a control group without mental disorders. Larger sample sizes would increase the power to detect DEPs, which might improve the precision of bioinformatics analysis. The lack of a control group made it difficult to interpret the direction of overexpression of proteins between the disorders. Second, this was a cross-sectional study, and thus a causal relationship between the protein profiles and disorders could not be determined. Moreover, in cross-sectional designs, the diagnosis of MDD always has the potential to be associated with undiscovered BD because of hypomanic/manic episodes that have not yet manifested or patient non-reporting. Third, our study lacked validation. Validation in a different group based on enzyme-linked immunosorbent assay would reveal the reliability of our results. However we were not able to confirm the results with alternative assays because of the insufficiency of residual samples. Fourth, as depressive symptoms were more severe in the MDD group, there are possibilities that such differences in depression severity could have influenced the main findings of the study. However, of note, the association between levels of the three major DEPs and diagnosis (MDD or BD) was still significant after additionally controlling for the total HAMD score as a covariate in linear regression models. Fifth, treatment histories could have influenced the results. Even though we only analysed those who were drug-free, treatment regimens and the total duration of treatment before the drug-free period could have influenced the results. Finally, other uncontrolled covariates such as exercise might have influenced the protein profiles. However, despite these limitations, our study’s strength is that we focused on the difference between MDD and BD, which is an understudied subject, and detected potential DEPs. Additionally, we only recruited drug-free subjects and performed sampling in the morning after overnight fasting, which contributed to the control of factors that affect protein expression.

## Conclusions

In conclusion, the present study demonstrated that serum proteomic profiles differed between MDD and BD. RAB7A, ROCK2, and XPO7 proteins were significantly changed after controlling for multiple comparisons. These proteins might enable differential diagnosis and expand our understanding of the pathophysiology of the two disorders. Additional studies with longitudinal designs, particularly to determine the longitudinal protein profiles from those who are initially diagnosed with MDD but develop hypomanic/manic symptoms later, are needed. Additionally, studies involving a larger sample size with more information on covariates that can influence the proteomic profiles, as well as those including validation designs, are warranted.

## Supplementary information

**Additional file 1.** List of 268 proteins subjected to statistical analysis. (Protein IDs and majority protein IDs).

**Additional file 2.** Major protein IDs and full protein IDs of the differentially-expressed proteins. (Protein IDs and majority protein IDs).

**Additional file 3.** IPA Canonical Pathway and IPA Diseases & Functions.

## Data Availability

The datasets used and/or analysed during the current study are available from the corresponding author on reasonable request.

## Abbreviations

MDD: Major depressive disorder
BD: Bipolar disorder
LC-MS/MS: Liquid chromatography-tandem mass spectrometry
HCD: Higher-energy collisional dissociation
FDR: False discovery rate
DDA: Data-dependent acquisition
DEP: Differentially expressed protein
RAB7A: Ras-related protein Rab-7a
ROCK2: Rho-associated protein kinase 2
XPO7: Exportin-7
LXR: Liver X receptor
RXR: Retinoic acid receptor
